# Identification of an ASC oligomerization inhibitor for the treatment of inflammatory diseases

**DOI:** 10.1038/s41419-021-04420-1

**Published:** 2021-12-13

**Authors:** Paula M. Soriano-Teruel, Guillermo García‑Laínez, María Marco-Salvador, Julián Pardo, Maykel Arias, Christian DeFord, Irmgard Merfort, María J. Vicent, Pablo Pelegrín, Mónica Sancho, Mar Orzáez

**Affiliations:** 1 Targeted Therapies on Cancer and Inflammation Laboratory, Centro de Investigación Príncipe Felipe, Valencia, Spain; 2 Polymer Therapeutics Lab., Centro de Investigación Príncipe Felipe, Valencia, Spain; 3 Centro de Investigaciones Biomédicas de Aragón (CIBA), Universidad de Zaragoza, Zaragoza, Spain; 4 Department of Pharmaceutical Biology and Biotechnology, Albert-Ludwigs-Universität, Freiburg, Germany; 5 Spemann Graduate School of Biology and Medicine (SGBM), Albert-Ludwigs-Universität, Freiburg, Germany; 6 Biomedical Research Institute of Murcia (IMIB-Arrixaca), University Clinical Hospital ‘Virgen de la Arrixaca’, Murcia, Spain

**Keywords:** Small molecules, Target validation

## Abstract

The ASC (apoptosis-associated speck-like protein containing a caspase recruitment domain (CARD)) protein is an scaffold component of different inflammasomes, intracellular multiprotein platforms of the innate immune system that are activated in response to pathogens or intracellular damage. The formation of ASC specks, initiated by different inflammasome receptors, promotes the recruitment and activation of procaspase-1, thereby triggering pyroptotic inflammatory cell death and pro-inflammatory cytokine release. Here we describe MM01 as the first-in-class small-molecule inhibitor of ASC that interferes with ASC speck formation. MM01 inhibition of ASC oligomerization prevents activation of procaspase-1 in vitro and inhibits the activation of different ASC-dependent inflammasomes in cell lines and primary cultures. Furthermore, MM01 inhibits inflammation in vivo in a mouse model of inflammasome-induced peritonitis. Overall, we highlight MM01 as a novel broad-spectrum inflammasome inhibitor for the potential treatment of multifactorial diseases involving the dysregulation of multiple inflammasomes.

## Introduction

ASC (apoptosis-associated speck-like protein containing a caspase recruitment domain (CARD)) is a protein of the innate immune system that participates in the formation of inflammasomes, which are macromolecular complexes responsible for the maturation and release of pro-inflammatory cytokines [1]. The inflammatory signaling engaged by inflammasome activation represents a rapid response that ensures the removal of detrimental stimuli and the repair of damaged tissue [2]. Therefore, inflammasome activation inhibits the spread of infection or the development of tumors and permits the development of an adaptive immune response [3]. However, inflammasome dysregulation has been implicated in the pathophysiology of conditions, such as Alzheimer’s disease [4, 5], diabetes [6], and cancer [7, 8], and in the development of inflammatory/autoimmune disorders, such as gout, silicosis, rheumatoid arthritis, genetically inherited periodic fever syndromes [9–11], and severe acute respiratory syndromes in response to viral infections [12].

In detail, ASC is an adaptor protein that bridges different sensor receptors for pro-inflammatory stimuli, mainly the nucleotide-binding domain and leucine-rich repeat-containing receptor (NLR) sensor proteins, with the zymogen procaspase-1 (pro-Casp-1) [1]. NLRs oligomerize in response to specific conserved microbial structures known as pathogen-associated molecular patterns or danger-associated molecular patterns [13]. NLR oligomerization promotes ASC binding and oligomerization in large filaments that aggregate into large structures known as ASC specks, thereby generating a multitude of pro-Casp-1 interaction sites that promotes the auto-processing and activation of pro-Casp-1 and, as a consequence, amplifying pro-inflammatory signaling [14, 15]. The ASC protein possesses two different protein domains, an N-terminal pyrin (PYD) domain (ASC^PYD^) and a C-terminal CARD domain (ASC^CARD^). ASC^PYD^ nucleates filaments and ASC^CARD^ links the oligomers to form specks [15–17]. Moreover, ASC^CARD^ interacts with pro-Casp-1^CARD^ to promote pro-Casp-1 oligomerization and activation and the subsequent processing and secretion of the mature forms of pro-inflammatory cytokines, such as interleukin (IL)-1β and IL-18 [18]. Of note, caspase-1 also cleaves Gasdermin D, which forms pores in the plasma membrane to trigger pyroptotic cell death [19–21]. ASC specks also possess an extracellular function that contributes to the spread of inflammatory signaling. ASC specks appear in the serum of cryopyrin-associated periodic syndrome patients [22] and in the cerebrospinal fluid of ischemic stroke model animal, where concentrations correlate with damage severity [23].

Several distinct inflammasome types have been described to date, with each differing with regards to the structure of the NLR component. The NLR family pyrin domain containing 1 (NLRP1), NLRP3, NLR family CARD domain-containing protein 4 (NLRC4), and interferon-inducible protein AIM2 inflammasomes are the most studied. The ASC dependence varies for each inflammasome, while pro-Casp-1 activation in NLRP3 and AIM2 inflammasomes requires ASC, the presence of ASC enhances pro-inflammatory signaling in CARD containing sensors, such as NLRP1 and NLRC4 [14, 15]. Recently, structural studies showed direct interaction between NLRP1^CARD^ and ASC^CARD^ and between NLRC4^CARD^ and ASC^CARD^ [16, 24]. As inflammatory responses depend on the activation of more than one inflammasome in several diseases [25–27], any drug that can inhibit numerous inflammasome complexes simultaneously would be of significant interest.

Currently reported inflammasome inhibitors target the oligomerization of NLR (inflammasome-specific inhibitors) [28], caspase-1 [29], and downstream events in the inflammasome activation cascade, such as IL-1β or its receptor [30], that compromise signaling events such as pyroptosis, which itself is responsible for the spread and amplification of inflammatory signaling. In this study, we identify MM01 as a modulator of inflammasome activity with a novel mechanism of action—the inhibition of ASC oligomerization and subsequent pro-Casp-1 activation. We demonstrate that MM01 disrupts ASC oligomerization triggered by different inflammasomes and inhibits downstream IL-1β/IL-18 release and pyroptosis in various cellular models of inflammation. Moreover, MM01 decreases neutrophil infiltration and pro-inflammatory cytokine accumulation in an in vivo model of peritonitis used as a proof of concept for the potential therapeutic capabilities of this novel inhibitor. Given the requirement for ASC in multiple inflammasome complexes, MM01 treatment may represent an effective treatment in a range of multifactorial diseases.

## Results

### MM01 blocks ASC-mediated pro-Casp-1 activation in vitro

**Fig. 1:**
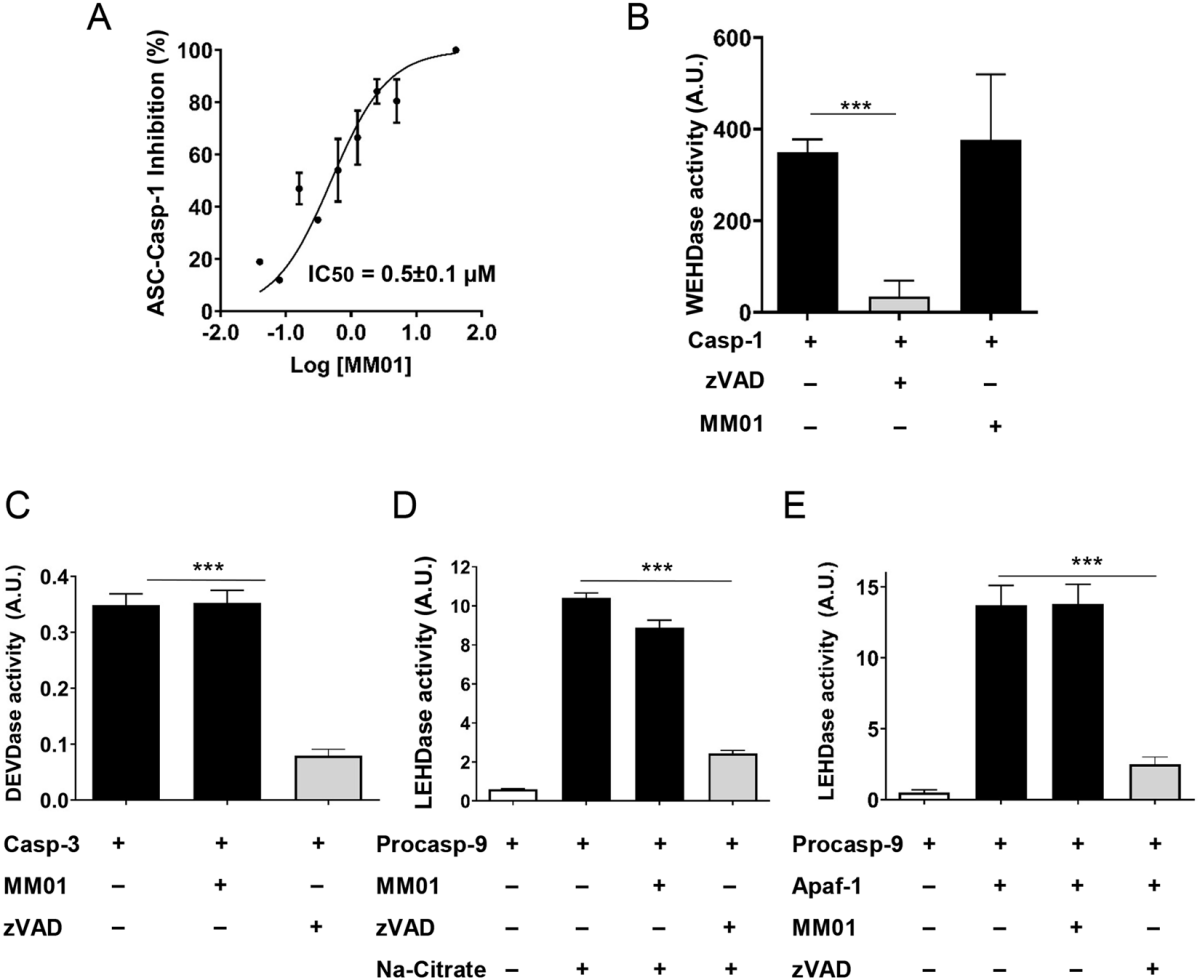
MM01 is an inhibitor of ASC-mediated pro-Casp-1 activation. **A** Dose–response curve of compound MM01 (up to 40 µM) from an in vitro ASC-mediated pro-Casp-1 activation assay. **B** Recombinant caspase-1 activity was monitored in the presence of MM01 (10 µM) to exclude compounds affecting active caspase-1. An inhibitor of the caspase catalytic site, zVAD (20 nM), was used as a control. **C** Recombinant caspase-3 activity was measured in the presence of zVAD (20 nM) and MM01 (5 µM). **D** Recombinant caspase-9 activity was measured in the presence of zVAD (20 nM) and MM01 (5 µM). As an activation control, procaspase-9 incubated with Na-citrate buffer was used. **E** Apoptosome activity was monitored after reconstitution in vitro in the presence of zVAD (20 nM) and MM01 (5 µM). In all cases, data represent the mean ± SD of three independent experiments (***p < 0.001).

To identify inhibitors of ASC oligomerization, we first screened for molecules that inhibited the activation of pro-Casp-1 by ASC in an in vitro assay using recombinant human ASC purified from *Escherichia coli* and pro-Casp-1 purified from *Baculovirus* (Fig. S1A). Details and development of the assay are summarized in Fig. S1A, B and in the “Materials and methods” section. Once ASC-dependent caspase-1 activation was established, we evaluated small-molecule compounds from the MyriaScreen Diversity Library I in dual-mixes and those active were deconvoluted to identify hit compounds that inhibited pro-Casp-1 by at least 60% (Fig. S1C, D). Positive hits were evaluated in a secondary screening against pre-activated caspase-1 to discard drugs targeting the active site of caspase-1 (Fig. S1E). To establish the best hit compound, we performed a drug-likeness score measured using the OSIRIS Property Explorer [31] (Fig. S1F). As a result of these assays, we identified MM01, a molecule that inhibited ASC-mediated pro-Casp-1 activation exhibiting an IC_50_ in the nanomolar range (Fig. 1A and Fig. S1G). We corroborated that MM01 do not targeted the active site of caspase-1, as opposed to the known caspase inhibitor zVAD (Fig. 1B). We also confirmed MM01 specificity via the absence of inhibition of other apoptotic caspases, such as caspase-3 or caspase-9 (Fig. 1C, D). To confirm the non-specific inhibitory activity of MM01, we also analyzed apoptosome inhibition. The apoptosome shares structural characteristics with the inflammasome and acts as a molecular platform for caspase-9 activation through CARD-CARD interactions. However, we did not observe any apoptosome inhibition in the presence of MM01 (Fig. 1E), confirming a specific mechanism of action for MM01 on the ASC-mediated pro-Casp-1 activation.

### MM01 prevents ASC oligomerization

**Fig. 2:**
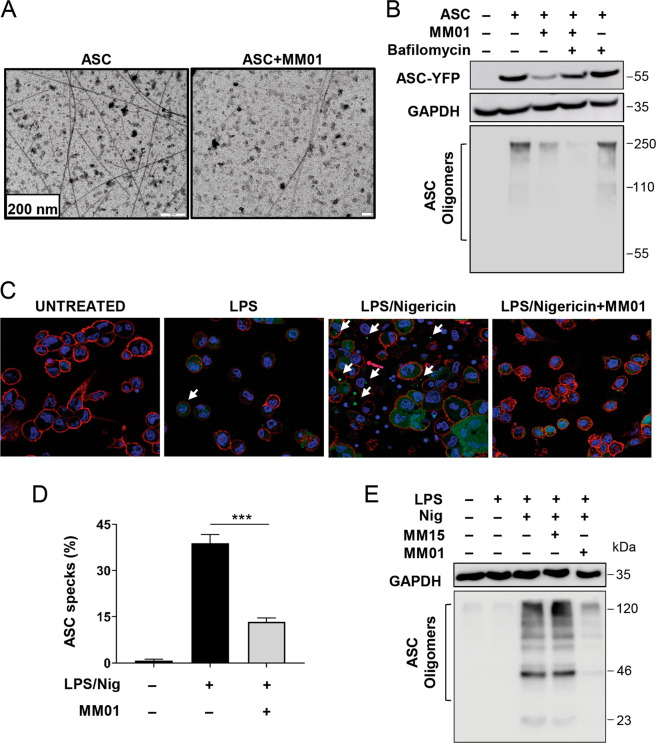
MM01 inhibits ASC oligomerization in vitro. **A** Electronic microscopic images of negatively stained preparations of human recombinant ASC filaments in the presence of MM01 (20 µM). **B** Western blot of lysates and crosslinked cytosolic pellets of ASC-YFP transfected HEK293 cells treated with MM01 (10 µM). GAPDH was used as a loading control for lysate samples. **C** Live-cell imaging of THP-1 ASC GFP cells treated with MM01 (10 µM) and stimulated with LPS (100 ng/ml) and nigericin (10 µM). The blue signal corresponds to DAPI staining and the red signal to the cytoplasmic marker WGA (wheat germ agglutinin). **D** Percentage of ASC specks in the THP-1 ASC GFP cells treated as in **C** and measured by flow cytometry. Data represent the mean ± SD of three independent experiments (***p < 0.001). **E** Western blot of crosslinked cytosolic pellets from PMA-differentiated THP-1 ASC GFP cells stimulated with LPS and nigericin and treated with the indicated compounds as in the above-described conditions.

We anticipated two possible mechanisms of action for MM01: the inhibition of ASC-pro-Casp-1 interactions or the interference of ASC oligomer formation that would lead to impaired recruitment and activation of pro-Casp-1. To explore the ability of MM01 to interfere in ASC oligomerization and speck assembly, we reconstituted ASC filaments in vitro from purified human recombinant ASC protein either in the presence or absence of MM01. Analysis by electron microscopy showed that the assembly of ASC specks was severely affected by the presence of MM01, thereby confirming the interference with ASC speck formation (Fig. 2A).

To corroborate the mechanism of action in the cellular milieu we transiently transfected ASC-YFP into HEK293 cells. HEK293 cells lack all the components of the inflammasome, and ASC-YFP transfection leads to spontaneous speck formation [32]. Interestingly in these assays, MM01 treatment avoided the formation of ASC oligomers (Fig. 2B); however, we observed that treatment with MM01 produced a decrease in ASC protein levels. The existence of a transcriptional regulatory effect of the MM01 compound was promptly discarded as no changes were observed in ASC mRNA levels, measured by quantitative reverse transcription polymerase chain reaction (Fig. S2A, B). Therefore, we hypothesized that MM01 could modify ASC to prevent oligomer formation and induce protein degradation. To confirm this hypothesis, and given the involvement of the lysosomal pathway in ASC degradation [33], we treated ASC-YFP-transfected HEK293 cells with bafilomycin, an inhibitor of autophagosome–lysosome fusion, alongside MM01 treatment. Bafilomycin treatment induced the recovery of ASC protein levels without the restoration of ASC-oligomerization following MM01 treatment, thereby confirming that MM01 interferes with ASC speck formation and facilitates ASC degradation in HEK293 cells (Fig. 2B).

Next, we studied the ability of MM01 to inhibit ASC speck formation using THP1-ASC-GFP cells, a cell line derived from THP-1 human monocytic cells that stably express an ASC-GFP fusion protein under the control of nuclear factor (NF)-κB-binding promoter. In resting cells, we did not observe any fusion protein; however, a priming signal (lipopolysaccharide (LPS) treatment) induces NF-κB-dependent ASC-GFP expression, while activation of the NLRP3 inflammasome by nigericin stimulation causes ASC-GFP speck formation. We monitored the ASC-GFP oligomerization upon treatment with both LPS and nigericin by confocal microscopy (white arrows in Fig. 2C) and observed a reduction of ASC speck formation following MM01 treatment (Fig. S2C). Quantification of ASC speck content by flow cytometry confirmed a >50% reduction of specks in MM01-treated cells (Fig. 2D).

To corroborate this observation, we chemically crosslinked THP-1 cell pellets and isolated ASC oligomers [34]. LPS and nigericin strongly induced ASC oligomerization, which was not altered following the treatment of cells with a negative control compound from the screening (MM15) (Fig. 2E). Indeed, only MM01 treatment strongly inhibited ASC oligomerization (Fig. 2E).

**Fig. 3:**
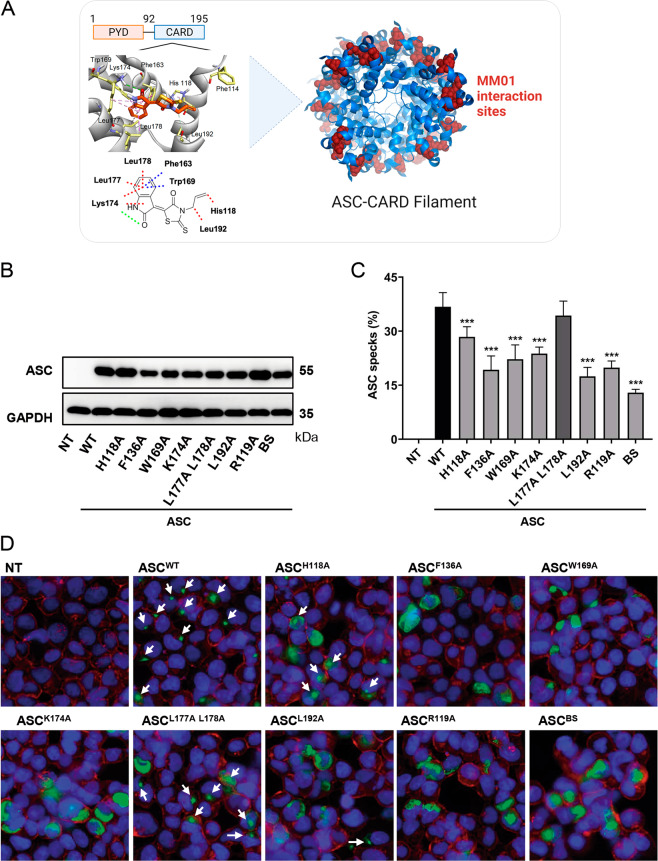
ASC-MM01-binding site characterization. **A** Docking model of ASC with MM01. Green lines represent hydrogen bonds, red lines show van der Waals interactions, and blue lines depict π interactions. Western blot (**B**) and percentage (**C**) of ASC specks in HEK293 cells transfected with the different ASC mutants and measured by flow cytometry. Data represent the mean ± SD of three independent experiments (***p < 0.001). **D** Live-cell imaging of HEK293 cells transfected with ASC mutants. The blue signal corresponds to DAPI staining and the red signal to the cytoplasmic marker WGA.

Docking studies with GOLD 5.2 (Protein Ligand Docking Software [35]) located critical interactions between ASC and MM01 involving residues His-118, Trp-169, Lys-174, Leu-177, Leu-178, and Leu-192 (Fig. 3A), which correspond to ASC^CARD^ domain. Mutations of residues Trp-169 or Arg-119 (near His-118) prompt the disruption of oligomerization [16]. Therefore, drugs such as MM01 may target these residues to impair oligomer formation and interfere with pro-Casp-1 activation.

To investigate the relevance of these residues in ASC speck formation, we mutated these positions by site-directed mutagenesis in the ASC-YFP construct to study ASC oligomer formation in HEK293. Site-directed mutagenesis rendered the ASC^H118A^, ASC^F136A^, ASC^W169A^, ASC^K174A^, ASC^L177A,L178A^, ASC^L192A^, and ASC^H118A,F136A,W169A,K174A, L177A-L178A,L192A^ (also called ASC^BS^) constructs. Additionally, mutated residue R119 was used as control because of its described inability to form specks [16]. HEK293 cells were transfected with these mutants and speck formation was analyzed by flow cytometry. Interestingly, with the exception of the more conservative mutations ASC[^L177A,L178A^], all other mutations produced a significant reduction in the number of ASC specks (Fig. 3C, D) without affecting protein expression (Fig. 3B), thereby indicating that the MM01 binding pocket is critical for ASC speck formation and represents a new site for therapeutic intervention.

### MM01 inhibits ASC-mediated inflammatory signaling

After demonstrating the molecular mechanism of action for MM01, we evaluated inflammasome inhibitory activity in THP-1 cells differentiated with phorbol 12-myristate 13-acetate (PMA) as a model of human macrophage function.

**Fig. 4:**
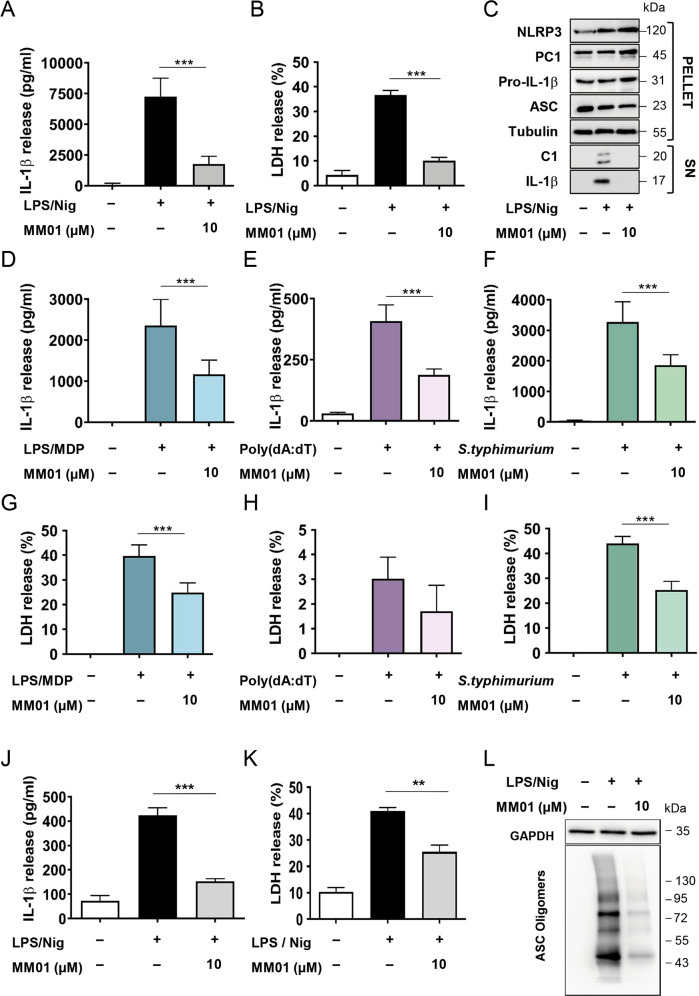
MM01 inhibits ASC-dependent inflammasome activity in vitro. **A** IL-1β secretion was evaluated by ELISA following activation of the NLRP3 inflammasome with LPS (100 ng/ml) and nigericin (Nig; 10 µM). Cells were treated with MM01 at 10 µM. **B** Measurement of LDH release into the extracellular medium under the above-described conditions. **C** THP-1 cells were stimulated as described above, and supernatants (SN) and pellets were analyzed by immunoblotting for IL-1β and cleaved caspase-1. A representative blot is shown. IL-1β measured by ELISA (**D**) and LDH release (**G**) were evaluated upon activation of the NLRP1 inflammasome with LPS (100 ng/ml) and MDP (50 µg/ml) in THP-1 cells. Cells were treated with MM01 at 10 µM. IL-1β secretion (**E**) and LDH release (**H**) were analyzed upon stimulation of the AIM2 inflammasome with poly (dA:dT) (200 μg/ml) in PMA-differentiated THP-1 cells treated or not with MM01 at 10 µM. IL-1β (**F**) and LDH secretion (**I**) were evaluated upon NLRC4 activation mediated by *Salmonella typhimurium* in THP-1 cells. Cells were treated with MM01 at 10 µM. MM01 inhibits LPS/Nig stimulation of isolated human PBMCs. IL-1β (**J**) and LDH (**K**) release were evaluated upon activation of the NLRP3 inflammasome with LPS (100 ng/ml) and nigericin (10 µM) and +/−MM01 at 10 µM in human PBMCs. **L** Western blot of crosslinked cytosolic pellets from PBMCs stimulated as described above. In all cases, asterisks represent significant differences to the stimulated control as determined by a one-way ANOVA with Tukey’s multiple comparisons test **p < 0.05; ***p < 0.001. All data expressed as mean ± SD of three independent experiments.

THP-1-derived-macrophages primed with LPS and activated by the specific NLRP3 activation stimulus nigericin exhibited an MM01-dependent reduction of IL-1β and IL-18 secretion (Fig. 4A and Fig. S3E). Interestingly, MM01 significantly reduced the levels of pyroptosis as measured by decreased lactate dehydrogenase (LDH) release (Fig. 4B). As pyroptosis contributes to the amplification of the pro-inflammatory signaling to neighboring cells [36], the MM01-mediated inhibition of pyroptosis could significantly dampen inflammatory spread. As expected, treatment with MM01 reduced the presence of active caspase-1 and processed IL-1β in cell supernatants without affecting the expression of other inflammasome components (Fig. 4C). We also confirmed the ability of MM01 to inhibit the NLRP3 inflammasome using another specific NLRP3-activating stimulus, such as ATP [37] (Fig. S3A–D). Thus, MM01, which interferes with ASC speck formation, inhibits NLRP3-dependent inflammasome activity.

Next, we evaluated the ability of MM01 to inhibit the activity of other ASC-dependent and ASC-enhanced inflammasome complexes. Treatment of THP-1 cells with LPS and muramyl dipeptide (MDP) activates the NLRP1 inflammasome [38]; under these conditions, MM01 pretreatment avoided pro-Casp-1 activation, IL-1β and IL-18 release, and protected cells from pyroptotic cell death (Fig. 4D, G and Fig. S4A, B). Cytosolic double-stranded DNA triggers the AIM2 inflammasome in PMA-differentiated THP-1 cells and induces IL-1β release. Due to the ASC dependence of this inflammasome, we also found that MM01 treatment significantly decreased IL-1β secretion (Fig. 4E, H). In the same line of evidence, MM01 treatment impaired IL-1β maturation and release following *Salmonella typhimurium*-induced NLRC4 activity [39] in THP-1 cells, leading to decreased pyroptosis (Fig. 4F, I and Fig. S4C).

Overall, these results confirm MM01 as an inhibitor of ASC oligomerization with the ability to inhibit the activity of a broad spectrum of inflammasomes.

### MM01 inhibits inflammasome activation in primary cultures

After demonstrating the activity of MM01 in established cell lines, we moved to evaluate MM01 in more physiological models of inflammation. We first evaluated the activity of MM01 in human peripheral blood mononuclear cells (PBMCs) stimulated with LPS and nigericin or ATP to activate the NLRP3 inflammasome. Encouragingly, treatment of these primary cultures with MM01 produced a significant decrease in IL-1β release and pyroptosis (Fig. 4J, K and Fig. S5A, B). ASC speck formation in this model is also compromised by the presence of the compound (Fig. 4L), strengthening our results.

**Fig. 5:**
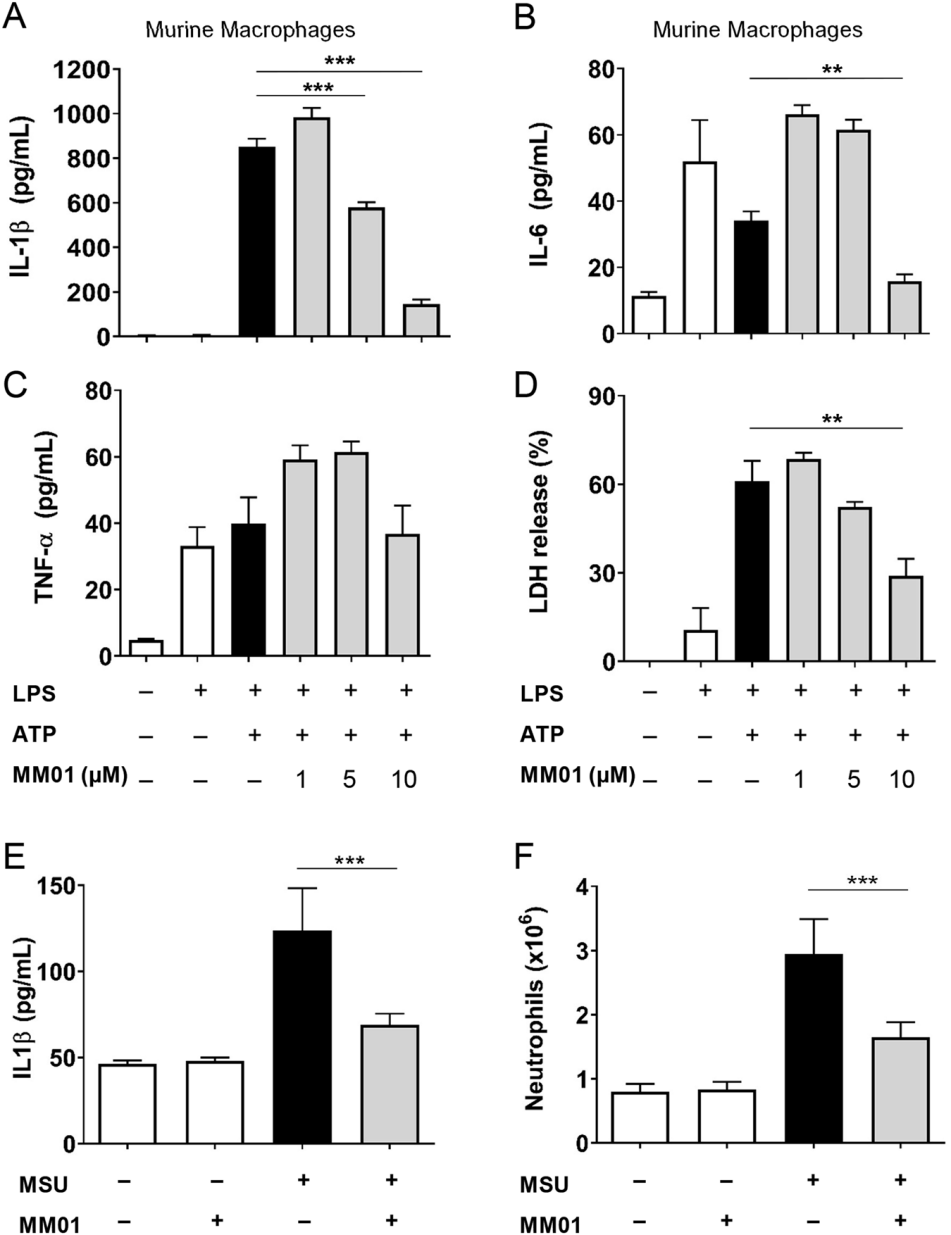
MM01 inhibits ASC-dependent inflammasome activity in stimulated murine peritoneal macrophages and in MSU-induced peritonitis mouse model. IL-1β (**A**), IL-6 (**B**), and TNF-α (**D**) secretion was evaluated by ELISA upon activation of the NLRP3 inflammasome with LPS (100 ng/ml) and ATP (2.5 mM) +/− MM01 at 1, 5, or 10 µM in isolated peritoneal macrophages. **C** Percentage of LDH release under the above-described conditions. In all cases, asterisks represent significant differences to the stimulated control (LPS/ATP), as determined by a one-way ANOVA with Tukey’s multiple comparisons test **p < 0.05; ***p < 0.001. All data expressed as mean ± SD of three experiments. **E** IL-1β detection by ELISA in the peritoneal cavity of C57BL/6 mice injected with MSU crystals with or without MM01 (10 mg/kg) treatment. **F** Neutrophil numbers in the peritoneal cavity of C57BL/6 mice treated as in the above-described conditions. Data are representative of two independent experiments (mean ± SD of *n* = 12). Asterisks represent significant differences to the stimulated control as determined by a one-way ANOVA with Tukey’s multiple comparisons test ***p < 0.001.

As we aimed to study the activity of MM01 in animal models of inflammation, we next confirmed the response of murine cells to MM01 treatment. For this purpose, we evaluated NLRP3 inhibition in murine peritoneal macrophages stimulated with LPS and ATP, with MM01 treatment significantly inhibiting both IL-1β levels and subsequent IL-6 release [40] (Fig. 5A, B). Importantly, we did not find a significant reduction in tumor necrosis factor-alpha (TNF-α) release (Fig. 5C), indicating the specific activity of MM01 as an inhibitor of inflammasome signaling. We also found a significant decrease in LDH release in MM01-treated murine macrophages (Fig. 5D), thereby confirming the inhibition of pyroptosis by MM01 in murine immune cells.

### MM01 inhibits NLRP3 activation in vivo

Finally, we evaluated the efficacy of MM01 in a murine mono-sodium urate (MSU)-induced model of peritonitis [41]. The particulate structure of MSU crystals produces a very potent activation of NLRP3 [42]. In this experimental model, the intraperitoneal injection of MSU crystals induces peritoneal inflammation, which can be followed by the increase in IL-1β levels and neutrophil infiltration in mice intraperitoneal fluid. The number of infiltrating neutrophils has been correlated with the extent of inflammasome activation [43]. Interestingly, treatment with MM01 (10 mg/kg) efficiently suppressed MSU-induced IL-1β production and peritoneal neutrophil recruitment (Fig. 5E, F), with no effect on IL-6 and TNF-α secretion (Fig. S5C, D), thereby providing evidence for the in vivo anti-inflammatory activity of the ASC speck inhibitor MM01.

## Discussion

In MM01, we describe a novel ASC inhibitor that avoids ASC speck formation and impedes pro-Casp-1 activation. Modulation of the inflammasome at the ASC level causes efficient inhibition of caspase-1 and cytokine release and significantly reduces pyroptotic cell death. Of note, MM01 treatment can inhibit inflammation both in vitro and in vivo in a mouse model.

While drugs that target IL-1β signaling, such as antibodies (canakinumab) or recombinant IL-1β receptor antagonists (anakinra), have found use as effective treatments for human inflammatory diseases, they do not cover all consequences of inflammasome activation, such as the additional pro-inflammatory cytokine release, the extracellular accumulation of ASC specks, or pyroptotic cell death, which all contribute to the spread of the pro-inflammatory signaling [44]. Our results reinforce the hypothesis that the perturbation of ASC interaction interfaces could represent a target for the development of broad-spectrum anti-inflammatory agents with improved cell recovery capabilities.

This inhibitor is predicted to act on a novel site on the ASC surface that impedes oligomerization, avoiding pro-Casp-1 recruitment and activation. The CARD domain of ASC is capable of forming filaments [16]. Amino acids involved in this interaction are close to those involved in the MM01 binding site. In particular, the W169 residue is involved in type II (hydrophobic) interactions that maintain the structure of said filament. R119, close to Histidine 118, is involved in type I interactions (charge-charge interactions) that stabilize the interaction between the helices of two ASC molecules. Further structural studies are needed to better understand how the named residues participate in oligomerization or structure stabilization. Here we have demonstrated that modulation at this level causes efficient inhibition of caspase-1 and cytokine release. Then, the results presented in this study validate the molecular target ASC and the drug, MM01, as a hit for further development into a lead compound. Of course, the activity of MM01 is limited to ASC-dependent inflammasomes, and in consequence some diseases where non-ASC dependent inflammasomes are involved will require the development of other specific drugs.

In line with our data, a related study has described the development of a single domain antibody fragment that specifically recognizes the CARD domain of human ASC [45]. This antibody impairs ASC (CARD) interactions, inhibits inflammasome activation, and protects cells from inflammatory cell death. Additionally, ASC inhibition by treatment with an IC-100 anti-ASC monoclonal antibody has prompted clear improvements in mice models of multiple sclerosis [46]. Recent research has also begun to describe ASC functions independent of the inflammasome. For example, the presence of ASC-deficient CD8+ T cells in transplanted mice decreases rejection responses, thereby suggesting a role for ASC in the modulation of cytotoxic T lymphocyte activity [47]. Thus, molecules targeting ASC such as MM01 could represent a new approach to inhibit transplant rejection. Interestingly, Schneider et al. recently established that ASC specks could activate Casp-8 and induce secondary pyroptosis in the absence of Casp-1 [36]. The inhibition of ASC by MM01 may effectively block secondary pyroptosis in this case.

Furthermore, growing evidence has implicated the involvement of numerous inflammasome types in human diseases. The activity of multiple inflammasomes has been implicated in inflammatory bowel disease in the regulation of commensal microbiota [48–50], while NLRP3, NLRP6, and NLRC4 expression can influence tumor formation [10]. Furthermore, the activity of at least two inflammasome types contribute to type 2 diabetes and other metabolic disorders [6, 51]. Both NLRP3 and NLRC4 have been described roles in neuroinflammation, with double knockouts shown to prompt improvements in astrogliosis and microglial accumulation [26]. While inflammasome inhibitors such as MCC950 [52] or CY-09 [53] have specific and highly effective anti-inflammatory effects on the activation of the NLRP3 inflammasome, conditions that involve more than one inflammasome type require a more general inhibitory strategy.

In light of the current circumstances, we note the involvement of multiple inflammasomes in viral infections [54]. Studies of severe acute respiratory syndrome coronavirus-2 indicate that virus severity correlates with an exacerbated inflammatory response, in which ASC specks play a fatal role [55]. Preclinical investigations with MM01 may contribute to a deeper understanding of viral infection mechanisms and offer novel effective therapeutic alternatives.

## Materials and methods

### Purification of procaspase-1 (pro-Casp-1)

Human His-tagged pro-Casp-1 was previously cloned into the pFastBac vector for *Baculovirus* expression. The expression plasmid was transformed into DH10Bac *E. coli* cells. Recombinant bacmids were then purified and used to transfect Sf9 insect cells. The virus stock was amplified and used to infect suspension cultures. Briefly, Sf9 cells were grown in 2 L of Grace’s Insect Medium (Gibco) and infected at 1,500,000 cells/ml with a recombinant virus that contained His-tagged pro-Casp-1 at a multiplicity of infection (MOI) of 1. Cells were then cultured for 16 h at 27 °C with constant gentle agitation to induce protein expression. Next, cells were centrifuged at 1000 × *g* for 10 min at 4 °C and washed with phosphate-buffered saline (PBS). The pellet was lysed with buffer lysis (100 mM HEPES-KOH pH 7.5, 50 mM KCl, 7.5 mM MgCl_2_, 5 mM NaEDTA, 5 mM NaEGTA, and protease inhibitors: Pepstatin, Leupeptin, and PMSF) using a Douncer and clarified by centrifugation (10,000 × *g*, 1 h, at 4 °C). The resulting supernatant that contained the recombinant protein was purified in a Ni-NTA (Ni2þ-nitrilotriacetate)-agarose column. After elution, pro-Casp-1 was further purified by ion-exchange chromatography (Mono Q) in 20 mM of HEPES-KOH pH 7.5, 10 mM KCl, 1.5 mM MgCl_2_, 1 mM EDTA, and 1 mM EGTA.

### Purification of ASC

ASC was previously cloned into the pET28a vector for expression in *E. coli*. Briefly, 8 L of LB medium, plus the antibiotics chloramphenicol and kanamycin, were inoculated with an overnight culture of freshly transformed His tagged-ASC-pET28a-BL21 (DE3) pLys *E. coli* (Invitrogen) and grown at 37 °C until optical density (OD) 0.7. ASC expression was induced with 0.7 mM isopropyl β-d-1-thiogalactopyranoside (IPTG) at 28 °C for 4 h. Cells were harvested (15 min, 6000 rpm at 4 °C), lysed in 300 ml of Buffer A (50 mM NaH_2_PO_4_, 300 mM NaCl, pH 8.0), and sonicated. After centrifugation at 11,000 rpm for 1 h at 4 °C, ASC was present in inclusion bodies (pellet). Pellets were then resuspended in 100 ml of denaturing Buffer B (50 mM NaH_2_PO_4_, 300 mM NaCl, 8 M urea, pH 8.0) and resonicated. Under these conditions, the His tagged-ASC protein was solubilized and purified using TALON resin (Qiagen). Re-folding was performed in the TALON resin at a gradient of decreasing urea concentrations, and the protein was finally eluted in 15 ml of Buffer C (50 mM NaH_2_PO_4_ pH 8.0, 300 mM NaCl, 500 mM imidazole). Desalting was performed in a PD-10 desalting column, and the protein was finally concentrated in a 10 K Amicon concentrator unit (Merck Millipore).

### Primary screening: ASC-mediated pro-Casp-1 reconstitution in vitro assay

Recombinant ASC (300 nM) was pre-incubated with the indicated concentration of compounds for 20 min at room temperature (RT) in Assay Buffer (20 mM HEPES pH 7.5, 10 mM KCl, 1.5 mM MgCl_2_, 1 mM EDTA, 1 mM EGTA, 1 mM DTT). Then pro-Casp-1 (50 nM) was added and incubated for another 10 min. Caspase-1 activation was measured using the Ac-WEHD-AFC fluorogenic substrate (80 µM; PeptaNova) by continuously monitoring the release of AFC in a Wallac Victor workstation. The MyriaScreen Diversity Library I® (Sigma-Aldrich) comprises 10,000 drugs dissolved in dimethyl sulfoxide (DMSO), selected to maximize the chemical diversity while maintaining drug-like properties. The compounds were evaluated at 20 µM with 1% of DMSO in dual-mixes for cost-effectivity reasons. Deconvolution took place by testing individually the compounds that made up each of the positive mixtures. Data are reported as the percentage of inhibition compared with the absence of the compound.

### Secondary screening: caspase-1 in vitro activation assay

Caspases have a high processivity rate. Base on this, active caspase-1 can be purified as indicated above for pro-Casp-1 recombinant protein but increasing infection time to 24 h. Once protein is active, activity can be monitored with a fluorogenic substrate. Recombinant caspase-1 (50 nM) was incubated with the indicated concentrations of compounds for 20 min at RT in Assay Buffer. Then Ac-WEHD-AFC fluorogenic substrate (80 µM) was added, and the activity was monitored in a Wallac Victor workstation. Compounds able to inhibit Casp-1 activity were discarded. Data are reported as the percentage of inhibition compared with the absence of the inhibitor.

### ASC filament formation in vitro

Recombinant human ASC protein was eluted from PD-10 column in an acid pH buffer to avoid precipitation. Then we induce filament formation achieving neutral pH by adding 3 M Tris buffer (pH 8.0) 1:5 ratio (vol/vol). ASC samples (12 µM) were incubated overnight at room temperature. A drop of 10 µl sample were placed on clean Parafilm and a mesh copper pure carbon coated grid were floated on top for 10 min. Then, the grids were transferred and contrasted with 1% uranyl acetate for 5 min. Excess fluid was removed and allowed to dry before examination with a transmission electron microscope FEI Tecnai G2 Spirit (ThermoFisher Scientific Company, OR, USA). All images were acquired using the Radius software with a digital camera Xarosa (EMSIS GmbH, Münster, Germany).

### Cell cultures

HEK293 cells (CRL-1573; ATCC) were grown in Dulbecco’s Modified Eagle Medium (DMEM)–high glucose medium supplemented with 10% fetal bovine serum (FBS). THP-1 (TIB-202; ATCC) cells were grown as supplier recommended. THP1-ASC-GFP were also cultured as supplier recommended (thp-ascgfp; InvivoGen). All cultures were maintained at 37 °C in a 5% CO_2_ atmosphere and were checked for mycoplasma contamination every 6 months.

### ASC speck assay

THP1-ASC-GFP cells were seeded at 1 × 10^6^ cells/ml the day before use in experiments on 35 mm glass-bottom culture dishes. The following day, the culture medium was replaced with fresh medium containing MM01 (10 µM) or vehicle for 30 min. Cells were then primed with 100 ng/ml LPS for 3 h and stimulated with 10 µM nigericin for 30 min. For confocal analysis, samples were fixed in 4% paraformaldehyde for 10 min at RT, washed several times, and prepared in mounting medium plus DAPI. Image acquisition used a Leica SP8 confocal microscope, and image processing was performed using the FiJi software. For cytometry analysis, samples were detached and analyzed in a CytoFlex Flow Cytometer (Beckman Coulter).

### ASC oligomerization assays

PMA-differentiated THP-1 cells were seeded at 1 × 10^6^ cells/ml in 6-well plates. Cells were treated with MM01 (10 µM) for 30 min, primed with 100 ng/ml LPS for 3 h, and then stimulated with 10 µM nigericin for 30 min. The supernatants were removed, cells were rinsed in ice-cold PBS and then lysed in NP-40 buffer (20 mM HEPES-KOH pH 7.5, 150 mM KCL, 1% NP-40 plus protease inhibitors). Lysates were centrifuged at 330 × *g* for 10 min at 4 °C. The pellets were washed and resuspended in PBS plus 2 mM disuccinimidyl suberate and incubated at RT for 30 min with rotation. Samples were then centrifuged at 330 × *g* for 10 min at 4 °C. The supernatant was removed, and the crosslinked pellets were then resuspended in sample buffer, boiled, and analyzed by immunoblotting.

HEK293 cells were seeded at 3.0 × 10^5^ cells/ml in 6-well plates and transfected with ASC-YFP construct [22] according to standard procedure for Lipofectamine 2000 reagent (Invitrogen). Then cells were treated with MM01 (10 µM) for 6 h and oligomers purified as described above. Bafilomycin A1 (InvivoGen) is a selective inhibitor of vacuolar-type H + ATPase (V-ATPase) that inhibits autophagosome and lysosome fusion. Bafilomycin (200 nM) treatment for 2 h was used as an inhibitor of protein degradation.

### ASC mutant analysis

ASC mutants in the active site predicted by MM01 molecular docking were generated by using the QuikChange Site-Directed Mutagenesis Kit (Agilent Technologies) in the ASC-YFP plasmid. These mutations include H118A, F163A, W169A, K174A, L192A, R119A, the double mutant L177A-L178A, and the binding site mutant (BS) containing all mentioned mutations. All molecular biology techniques were performed according to the standard procedures.

To assess the effect of ASC mutants on speck formation, HEK293 cells were seeded at a concentration of 4.0 × 10^5^ cells/ml in 12-well plates. The next day, Lipofectamine^TM^ 2000 (Invitrogen) was used according to the manufacturer’s instructions to transfect the cells with the ASC-YFP plasmid. After 24 h, HEK293 cells were harvested to analyze the speck formation by flow cytometry and confocal microscopy as well as the oligomerization formation by chemical crosslinking. In addition, whole-cell extracts were obtained to check the presence of the fusion protein in every sample.

### Docking calculations with GOLD 5.2

The crystal structure of human ASC (PDB ID: 2KN6) was downloaded from the protein data bank2 and subjected to docking calculations using the GOLD 5.2 software (CCDC, Cambridge, UK). The internal energy of the compounds was first minimized using the MM2 protocol and submitted to GOLD in SDF format. Docking experiments were performed three times using the default docking settings and ChemScore as the scoring function. A total of 30 genetic algorithms (GA) runs were set for each compound. To accelerate the calculations, the program was allowed to stop the GA runs when the top three solutions were within 1.5 Å root mean square deviation. Intermolecular interactions were described using Discovery Studio 4.0 (Accelrys Inc., San Diego, CA, USA).

### Cellular assay for inflammasome activation

The molecules were evaluated in THP-1 or PMA-differentiated THP-1 cells (50 nM PMA for 24 h) stimulated with LPS (100 ng/ml) and MDP (50 μg/ml) or with LPS plus adenosine triphosphate (2.5 mM ATP) to stimulate ASC-dependent inflammasomes NLRP1 and NLRP3, respectively. Briefly, 1 × 10^6^ cells were seeded in 6-well plates in 1 ml RPMI media that contained 1% FBS. Cells were either mock-treated or primed with compounds at the indicated concentrations for 30 min, followed by treatment with LPS (Sigma) and MDP (Sigma) or ATP (Sigma) for 6 h at 37 °C. In the case of nigericin stimulation, cells were primed with LPS for 3 h, and then 10 µM nigericin (Sigma) was added for 30 min. Treatment with 10 µM MM01 was optimal in cellular assays due to the intrinsic characteristics of the compound in the cellular environment. Supernatants were harvested and clarified by centrifugation at 1500 rpm at RT, and cytokine analysis was then performed. To induce NLRC4 inflammasome, cells were pretreated with the compounds for 30 min, and then cells were stimulated for 3 h with *Salmonella Typhimurium* (MOI 5). To induce AIM2 inflammasomes, cells were pretreated in the same conditions as before and transfected with poly(dA:dT) (0.5 μg/ml; Sigma) overnight using Lipofectamine 2000 (Invitrogen).

Both IL-1β secretion and the release of IL-18 were monitored by enzyme-linked immunosorbent assay (ELISA; 557953 BD OptEIA™ and BMS267-2 from eBioscience) following the manufacturer’s instructions. Cell viability was analyzed in parallel by evaluating the release of LDH according to a commercial kit (CytoTox-ONE™ Homogeneous Membrane Integrity Assay G7890; Promega). The release of LDH was calculated using the formula: the release of LDH (%) = 100 × (Abs490 treated − Abs490 untreated cells)/Abs490 untreated cells lysed with Triton 9% (maximum release of LDH).

### Murine macrophage and PBMC assays

Human PBMCs were purchased from Lonza (CC-2702). PBMCs were plated in 24-well plates at 5,000,000 cells/well in serum-free RPMI medium. Cells were primed for 3 h with 200 ng/ml pure LPS followed by 30 min with 10 µM MM01 and 1 h with 10 µM nigericin. Supernatants were harvested and clarified by centrifugation at 1500 rpm at RT, and cytokine and caspase-1 activation analysis was performed as previously described.

Mice (BALB/c) were euthanized, and 5 ml of cold medium was injected (20 gauge needle) into the peritoneal region. The aspirated fluid from the peritoneum was centrifuged for 10 min at 400 × *g* and the cell pellet was dissolved in cold DMEM/F12-10 (1 ml). Isolation of macrophages was performed according to a previously described protocol [56].

Macrophages were plated in 24-well plates at 400,000 cells/well overnight. Cells were primed for 3 h with 200 ng/ml pure LPS (Alexis Biochemicals Corporation) in serum-free IMDM media followed by 30 min with the compounds and 5 mM ATP. Supernatants were harvested and clarified by centrifugation at 1500 rpm at RT, and cytokine and caspase-1 activation analysis was performed as previously described for the THP-1 cell line.

### Immunoblotting

The supernatants of the treated cells were precipitated by the chloroform–methanol method, as previously described [34]. Pellets were obtained by lysing cells in 25 mM of Tris-HCl pH 7.4, 1 mM EDTA, 1 mM EGTA, and 1% sodium dodecyl sulfate (SDS), plus protease and phosphatase inhibitors. The protein concentration was determined by the BCA protein assay. Samples were separated in a 14% SDS-polyacrylamide gel electrophoresis gel, transferred to a nitrocellulose membrane, and blocked with 5% skimmed milk for 1 h. Then the membrane was incubated overnight with primary antibodies: α-casp1 (1:1000; Cell Signaling 2225), α-IL-1β (1:1000; Acris R1130P), α-NLRP1 (1:1000; ALX-210-904-R100), α-NLRP3 (1:1000; Cell Signaling 15101), α-ASC (1:1000; Santa Cruz Biotechnology sc-22514-R), α-gapdh (1:1000; Cell Signaling 2118), and α-tubulin (1:3000; Abcam ab6160) at 4 °C. Membranes were washed and probed with the appropriate secondary antibody conjugated with peroxidase for enhanced chemiluminescence detection (Amersham Pharmacia Biotech).

### MSU-induced peritonitis mouse model

In vivo model was performed as described in Bioprotocols [41] using an approach approved by the Institutional Animal Ethical Committee (CEEA 2018_28). C57BL/6 mice (9 weeks) were injected intraperitoneally with 10 mg/kg MM01 or vehicle (PBS + 0.1% DMSO) 30 min before intraperitoneal injection of MSU (2 mg MSU crystals in 200 µl sterile PBS). After 6 h, mice were sacrificed, and peritoneal lavage with 5 ml of PBS was performed. Cytokine IL-1β (DY401 R&D Systems), TNF-α (555268 BD OptEIA™ Set), and IL-6 (555240 BD OptEIA™ Set) secretion was determined by ELISA, and neutrophil content measured by flow cytometry in ACVLAB Laboratorio Valencia. A total of *N* = 12 animals per condition was used. Groups were distributed randomly without any specific methodology. No blinding was done. Experiment was done two times, 50% males and 50% females. Sample size was calculated using the GRANMO Software (https://www.imim.es/ofertadeserveis/software-public/granmo).

### Statistical analysis

Data are expressed as mean ± SD and the number of biological replicates is indicated in each figure legend. Data were analyzed with the GraphPad Prism software. Statistical significance (**p < 0.05; ***p < 0.01) was assessed by Student’s *t* test when used to compare two groups. When comparing more than two groups, a one-way analysis of variance test with Tukey’s multiple post test comparisons was used to identify differences.

## Supplementary information

Supplemental Information

Quality checklist

## Data Availability

All relevant data are within the manuscript and its Supplementary Information files.
