# Supplementary material for: Identification of an ASC oligomerization inhibitor for the treatment of inflammatory diseases
Source: Cell Death Dis. 2021 Dec 13;12(12):1155. doi: 10.1038/s41419-021-04420-1 (PMC8667020; doi:10.1038/s41419-021-04420-1)
Supplement: Supplementary file 1 — Supplemental Information [file 41419_2021_4420_MOESM1_ESM.pdf]

Supplementary Figure 1

A

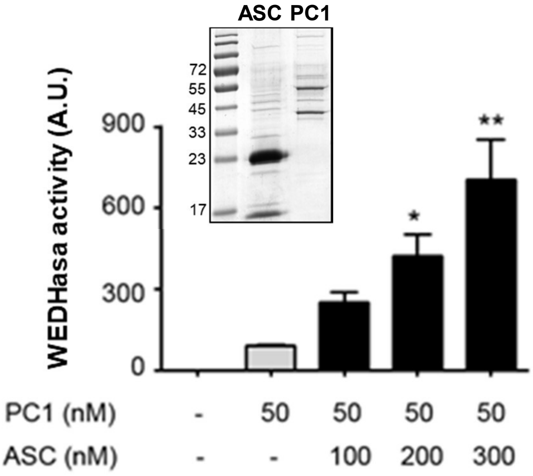

B

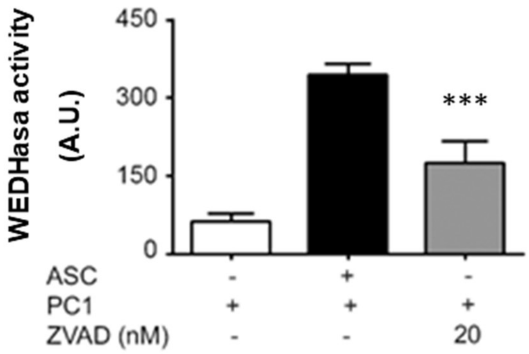

C

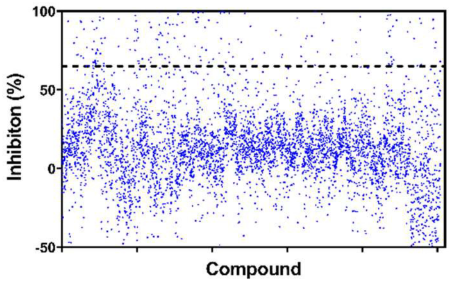

D

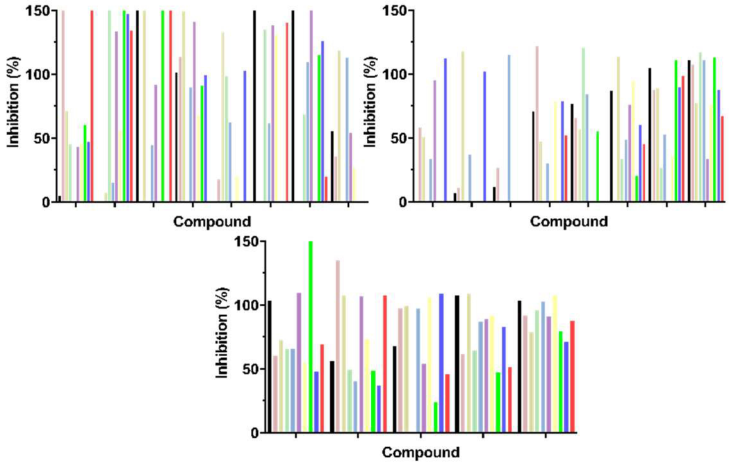

E

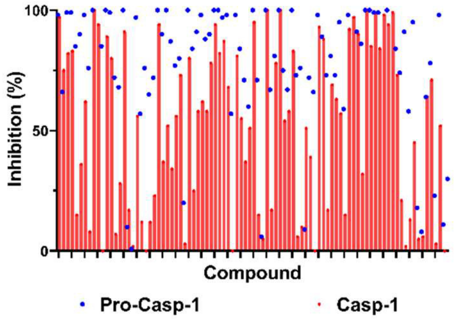

F

CODE	IC50 (µM)	% INH C1	OSIRIS
MM01	0,5±0,1	17±15	0,84
MM02	0,4±0,2	14±13	0,89
MM03	0,2±0,1	15±18	0,55
MM04	0,3±0,1	12±12	0,42
MM05	0,5±0,1	0±0	0,11
MM06	1,5±0,8	2±4	0,77
MM07	3,7±0,9	26±12	0,6
MM08	3,9±0,7	12±14	0,32
MM09	4,4±0,6	25±17	0,41
MM10	7±2	7±10	0,27
MM11	11±3	8±10	0,75
MM12	22±5	10±10	0,78
MM13	27±7	0±0	0,21
MM14	37±10	17±19	0,09

G

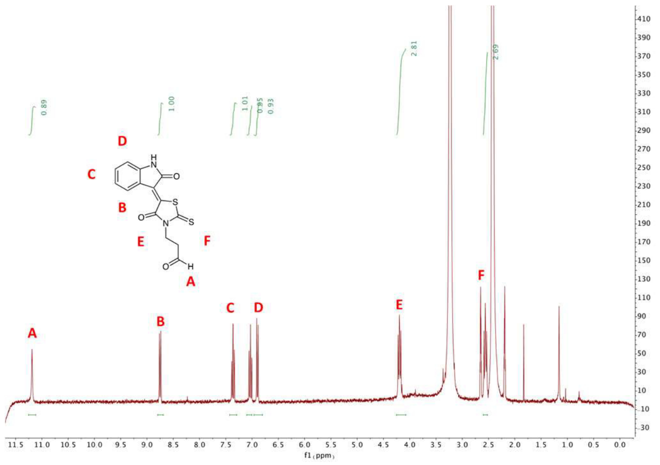

Supplementary Figure 2

A

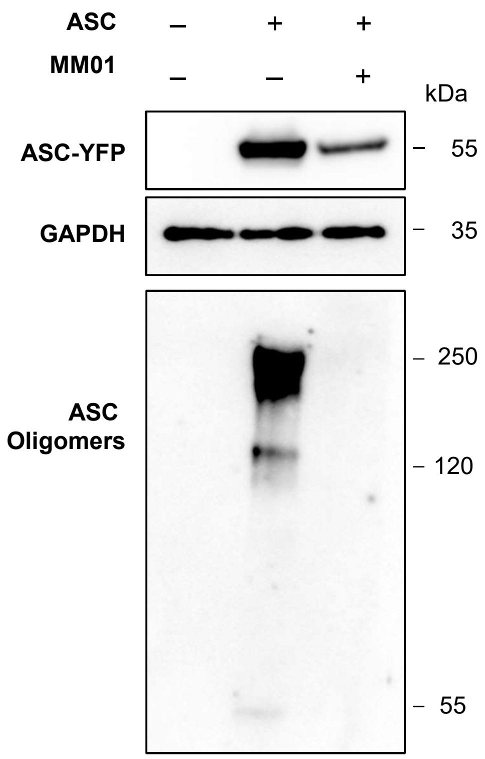

B

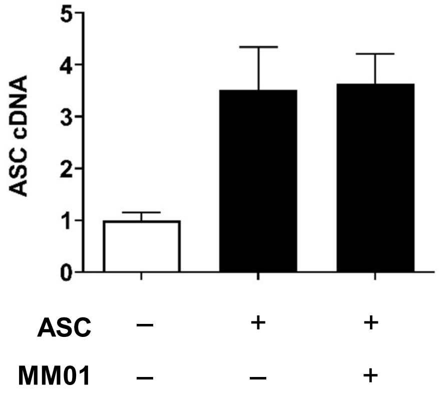

C

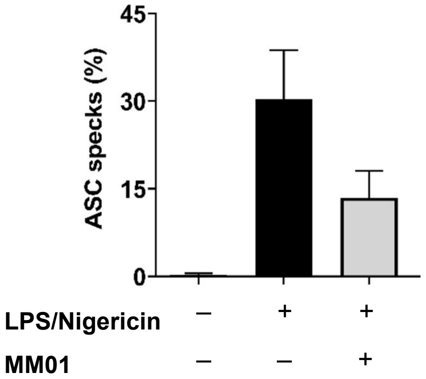

Supplementary Figure 3

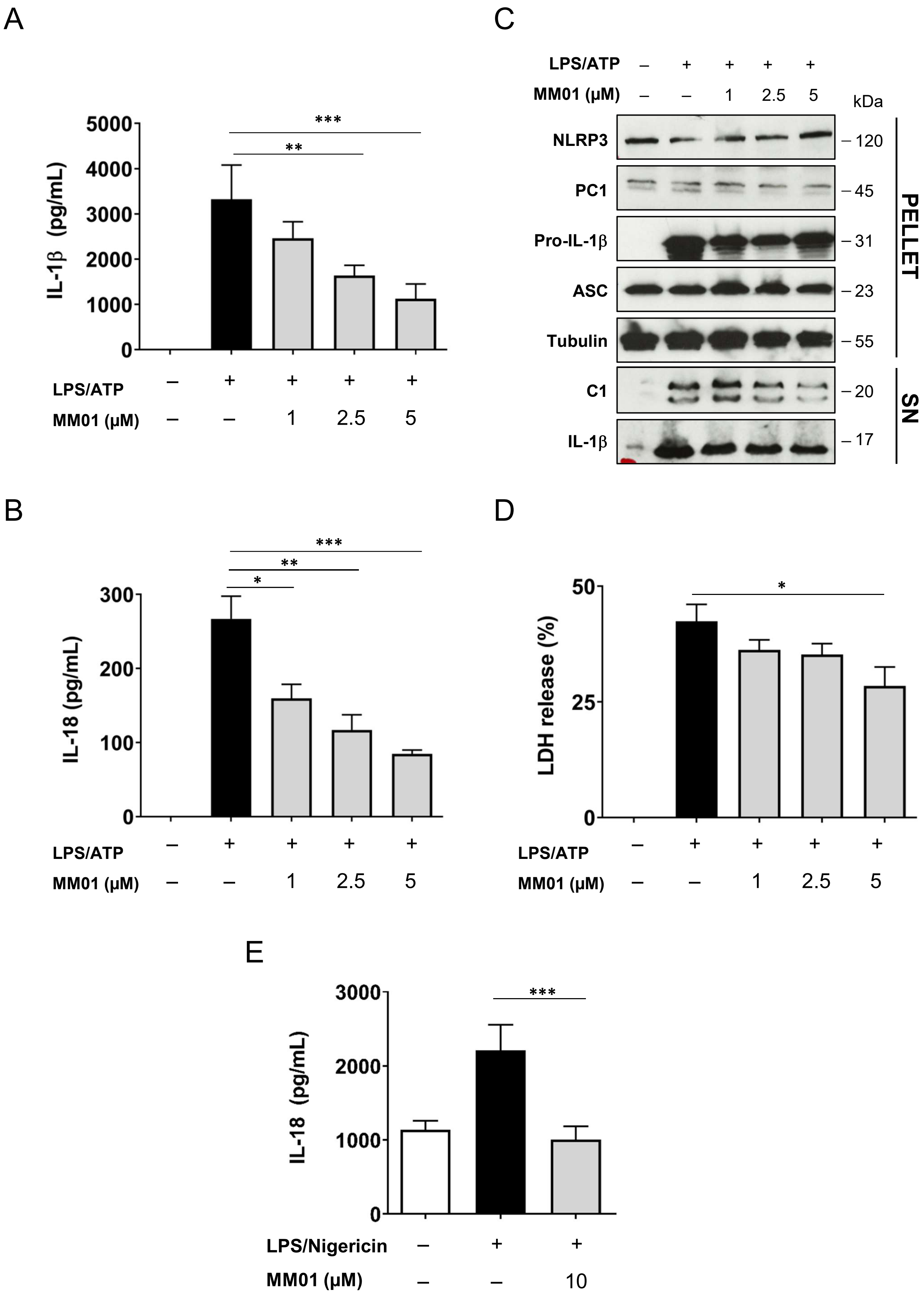

Supplementary Figure 4

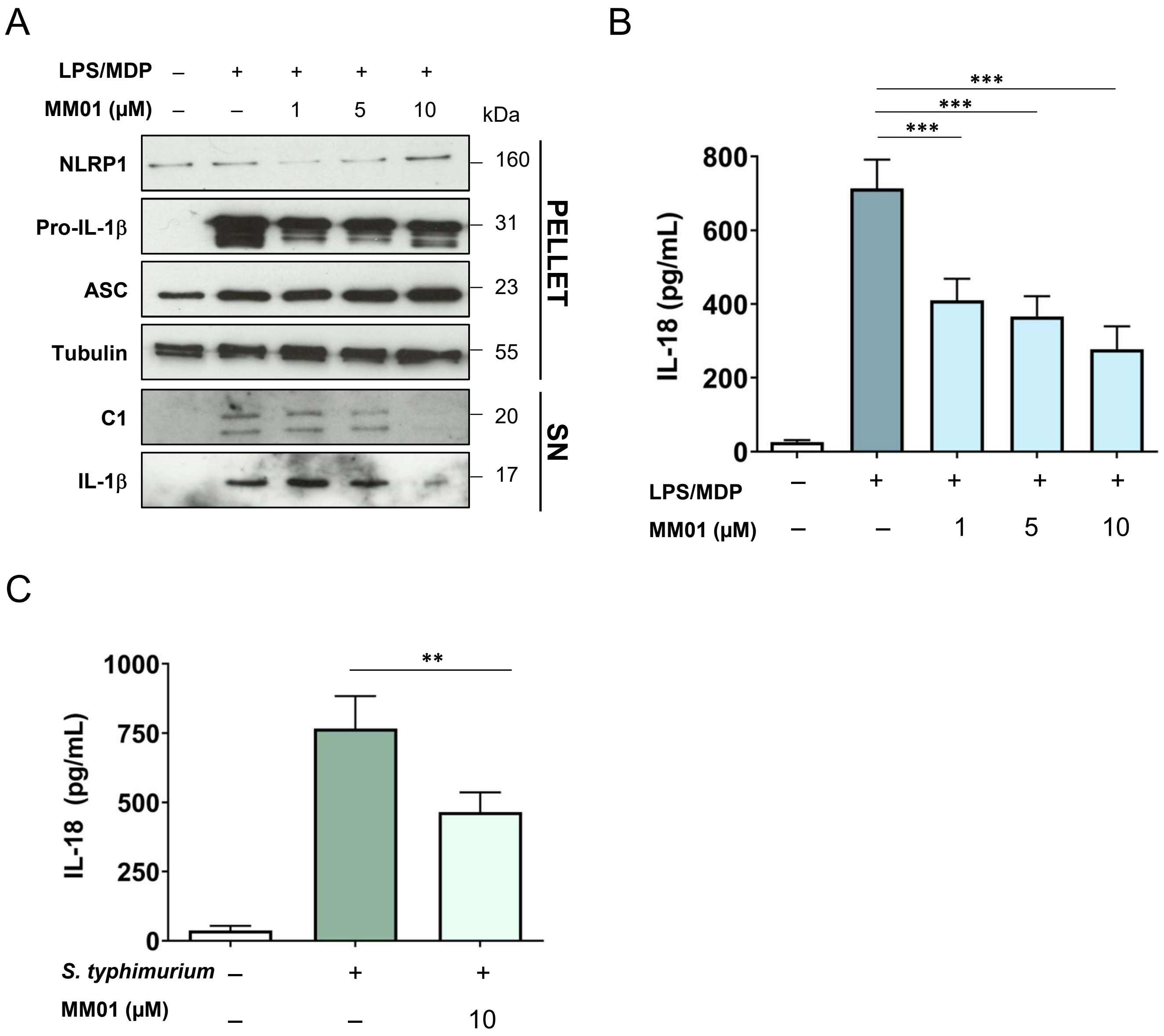

Supplementary Figure 5

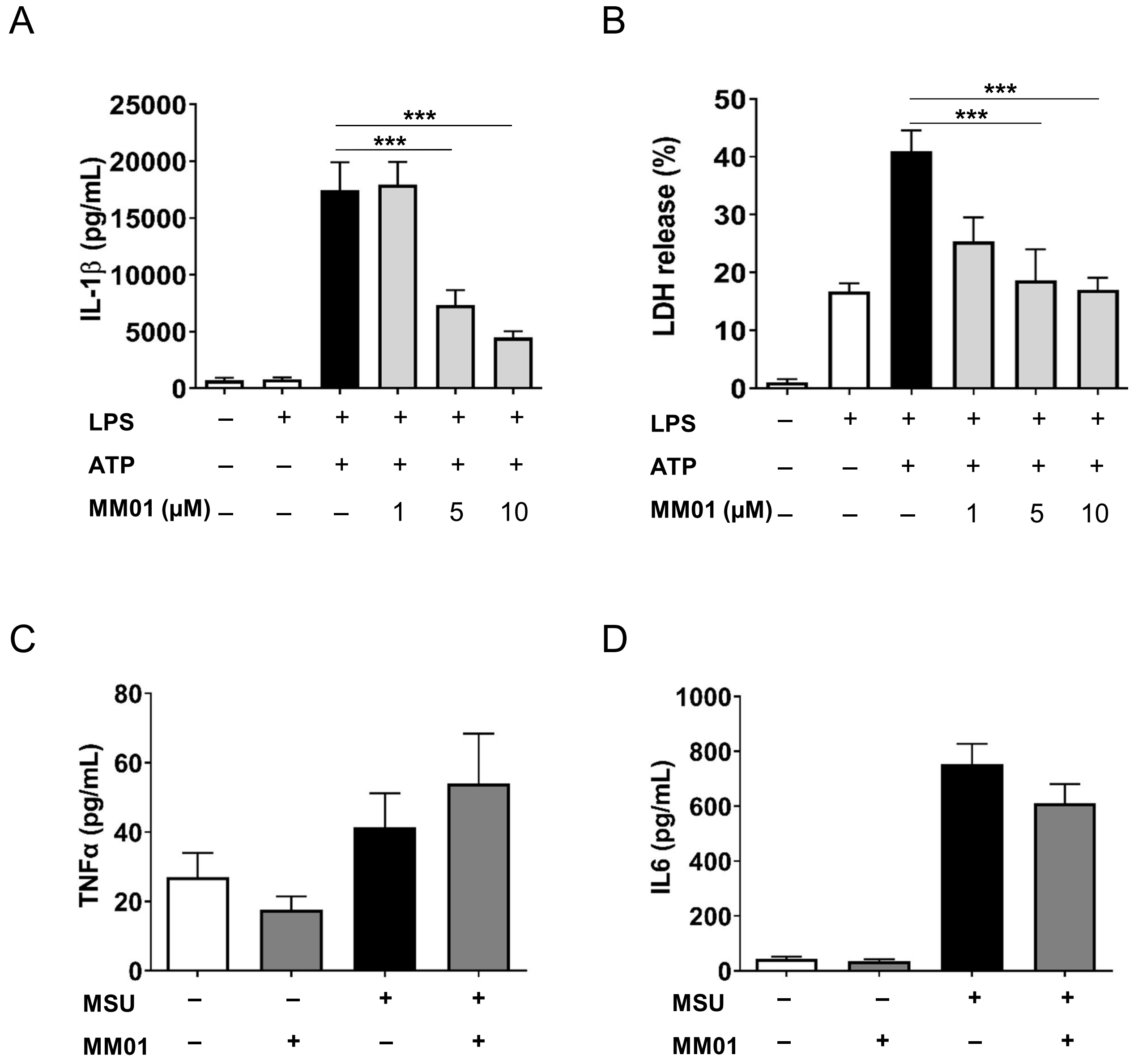

Supplementary Figure Legends

Fig. S1 Assay optimization for high-throughput screening. A Coomassie-stained gel for purified recombinant ASC (ASC) and pro-Casp-1 proteins (PC1) in the upper panel. ASC-mediated pro-Casp-1 activation assay (graph). Caspase-1 activation in the presence of different concentrations of recombinant ASC measured by the release of the AFC (7-Amino-4-trifluoromethylcoumarin) fluorescent probe conjugated to a caspase-1 specific substrate (WEDH peptide). A.U. means arbitrary units. **B** Substrate monitoring of the control screening conditions. Basal activation of recombinant pro-Casp-1 (PC1) alone, in the presence of recombinant ASC, and treated with zVAD as a caspase inhibitor. **C** Dot plot representation of primary screening for 5,000 dual-mixes. Percentage of inhibition of pro-Casp-1 activation according to the above described assay (see details in Methods section). Threshold was stablished in 60% of inhibition. **D** Graphical representation of deconvolution assay. Percentage of ASC-mediated pro-Casp-1 inhibition of the individual compounds selected from the positive mixes. **E** Secondary screening of the positive hits against pre-activated Casp-1 to discard compounds targeting the active site of Casp-1. Graphical representation of percentage of inhibition for both, primary (blue dot) and secondary (red bar) assays, of each positive hit. **F** Summary of properties of selected compounds (20 µM) able to inhibit pro-Casp-1. IC_{50} values, percentage of Casp-1 inhibition (INH C1), and drug-likeness measured by OSIRIS Property Explorer were calculated for the 14 selected hits. The prediction process in OSIRIS relies on a pre-computed set of structural fragments created from all compounds of the RTECS (registry of toxic effects of chemical substances) database that give rise to toxicity alerts in case they are encountered in the structure drawn. **G** ^{1}H NMR spectra of compound MM01 in deuterated DMSO (300 MHz). δ: 11.19 (s, 1H), 8.75 (dt, J = 7.8, 1.1 Hz, 1H), 7.36 (d, J = 1.3 Hz, 1H), 7.03 (d, J = 1.1 Hz, 1H), 6.96 – 6.80 (m, 1H), 4.20 (s, 3H), 2.56 (dd, J = 8.5, 7.2 Hz, 3H).

Fig. S2 MM01 inhibits ASC oligomerization. A Immunoblot analysis of ASC expression and ASC oligomerization in lysates and crosslinked cytosolic pellets of ASC-YFP transfected HEK293 cells treated with MM01 (10 µM). GAPDH used as a loading control for lysate samples. **B** RT-qPCR of ASC expression in the above described conditions. Data expressed as the fold change of ASC gene expression normalized to GAPDH compared

to non-transfected and untreated sample. **C** Quantification of confocal images of THP-1 ASC GFP cells treated with MM01 (10 µM) and stimulated with LPS (100 ng/mL) and nigericin (10 µM). Graph represents percentage of ASC specks in ten distinct images taken for each condition in three independent experiments.

Fig. S3 MM01 inhibits LPS and ATP-mediated NLRP3 activation in THP-1 cells. IL-1β **(A)** and IL-18 **(B)** secretion were evaluated by ELISA upon activation of the NLRP3 inflammasome with LPS (100 ng/ml) and ATP (2.5 mM). Cells were treated with MM01 at 1, 2.5, or 5 µM. **C** In these conditions, supernatants (SN) and pellets were also analyzed by immunoblotting for IL-1β and cleaved caspase-1. A representative blot is shown. **D** Measurement of LDH release into the extracellular medium under the above-described conditions. Asterisks represent significant differences to the stimulated control (LPS/ATP), as determined by a one-way ANOVA test with Tukey's multiple post-test comparisons **$p<0.05$; ***$p<0.001$. All data expressed as the mean±SD of three experiments. **E** IL-18 secretion was evaluated by the ELISA technique upon activation of the NLRP3 inflammasome with LPS (100 ng/ml) and nigericin (10 µM). Cells were treated with MM01 at 10 µM.

Fig. S4 MM01 inhibits NLRP1 and NLRC4 inflammasome activation. A THP-1 cells activate NLRP1 inflammasome upon treatment with LPS (100 ng/ml) and MDP (50 µg/ml). Cells were treated with MM01 at 1, 5, or 10 µM. Supernatants and inputs were analyzed by immunoblotting for IL-1β and cleaved caspase-1. A representative blot is shown. **B** IL-18 secretion, measured by ELISA, was evaluated in the above described conditions in THP-1 cells. Asterisks represent significant differences to the stimulated control (LPS/MDP), as determined by a one-way ANOVA test with Tukey's multiple post-test comparisons **$p<0.05$; ***$p<0.001$. All data expressed as the mean ± SD of three experiments. **MM01 inhibits NLRC4 activation mediated by *Salmonella typhimurium* in THP-1 cells. C** IL-18 secretion was evaluated by ELISA upon activation of the NLRC4 inflammasome in THP-1 cells stimulated as described in Materials and Methods. Cells were treated with MM01 at 10 µM. Asterisks represent significant differences to the stimulated control as determined by a one-way ANOVA test with Tukey's multiple post-test comparisons **$p<0.05$; ***$p<0.001$. All data expressed as the mean ± SD of three experiments.

Fig. S5 MM01 inhibits LPS/ATP stimulation of isolated human PBMCs. IL-1β (**A**) and LDH (**B**) release were evaluated upon activation of the NLRP3 inflammasome with LPS (100 ng/ml) and ATP (2.5 mM), +/- MM01 at 1, 5, or 10 µM in human PBMCs. Asterisks represent significant differences to the stimulated control as determined by a one-way ANOVA with Tukey's multiple comparisons test ***$p<0.001$. **MM01 does not inhibit IL-6 and TNF-α secretion in MSU-induced peritonitis mouse model**. ELISAs for IL-6 (**C**) and TNF-α (**D**) in the peritoneal cavity of C57BL/6 mice injected with MSU crystals with or without MM01 (10 mg/kg) treatment. Data are representative of two independent experiments (mean and SD of n=12).

Supplementary Materials and Methods

Other recombinant proteins: caspase-3, procaspase-9 and Apaf-1

Overexpression and purification of His-tagged caspase-3 (Casp-3) and procaspase-9 (Procasp-9) was developed as previously reports[1, 2, 3]. rApaf-1 was obtained from a baculovirus expression system as was described previously[4].

Caspases activation assays

Caspase-3 activity in vitro was measured as described in Woolan et al.[3]. Briefly, caspase-3 was incubated 10 min at 50 nM in the following reaction buffer: 50 mM HEPES pH7.4, 50 mM KCl, 0.1 mM EDTA, 1 mM DTT and 0.1% CHAPS. Activity was monitored by adding 20 µM Ac-DEVD-afc for 5 min and reading fluorescence in a Victor 2 spectrofluorimeter (λexc= 390 nm; λem= 510 nm).

Caspase-9 was activated by effect to kosmotropic salts, in particular using Na-citrate as previously reported[5]. The protein at final concentration of 100 nM was pre-activated in SC buffer (50 mM $Na_2HPO_4 \cdot 7H_2O$, 150 mM NaCl, 1.5% Sucrose, 0.05% CHAPS, 10 mM DTT, 0.7 M Sodium Citrate Tribasic, pH 7.4) at RT during 20 minutes. Then the reaction was developed in buffer reaction S, maintaining the final concentration of SC buffer components except the Na-citrate which was diluted at final concentration of 35 mM, during 20 min at RT. Enzyme activity was monitored by Ac-LEHD-afc substrate (50µM) using a Victor 2 spectrofluorimeter (λexc= 390 nm; λem= 510 nm).

In vitro Apoptosome Reconstitution Assay

Assay was performed incubating purified rApaf-1 (150 nM) in Buffer A (20 mM HEPES, 10 mM KCl, 1.5 mM $MgCl_2$, 1 mM EDTA, 1 mM EGTA, 1 mM DTT, 0.1 mM PMSF) at 30ºC for 15 min. Then, 1 µM dATP and 1 nM Cyt c were added and incubated at 30ºC for 40 min. 100 nM Caspase-9 was added and activity was monitored adding Ac-LEHD-afc substrate as described above.

References Supplementary Info

1. Riedl SJ, Li W, Chao Y, Schwarzenbacher R, Shi Y. Structure of the apoptotic protease-activating factor 1 bound to ADP. *Nature* **434**, 926-933 (2005).

2. Stennicke HR, Deveraux QL, Humke EW, Reed JC, Dixit VM, Salvesen GS. Caspase-9 can be activated without proteolytic processing. *J Biol Chem* **274**, 8359-8362 (1999).

3. Wolan DW, Zorn JA, Gray DC, Wells JA. Small-molecule activators of a proenzyme. *Science* **326**, 853-858 (2009).

4. Malet G, Martin AG, Orzaez M, Vicent MJ, Masip I, Sanclimens G*, et al.* Small molecule inhibitors of Apaf-1-related caspase- 3/-9 activation that control mitochondrial-dependent apoptosis. *Cell Death Differ* **13**, 1523-1532 (2006).

5. Boatright KM, Renatus M, Scott FL, Sperandio S, Shin H, Pedersen IM*, et al.* A unified model for apical caspase activation. *Mol Cell* **11**, 529-541 (2003).
